# Synthetic Aβ peptides acquire prion-like properties in the brain

**DOI:** 10.18632/oncotarget.2819

**Published:** 2014-11-25

**Authors:** Xiangzhu Xiao, Ignazio Cali, Jue Yuan, Laura Cracco, Paul Curtiss, Liang Zeng, Mai Abouelsaad, Dimitris Gazgalis, Gong-Xian Wang, Qingzhong Kong, Hisashi Fujioka, Gianfranco Puoti, Wen-Quan Zou

**Affiliations:** ^1^ Department of Pathology, Case Western Reserve University, Cleveland, Ohio, USA; ^2^ National Prion Disease Pathology Surveillance Center, Case Western Reserve University, Cleveland, Ohio, USA; ^3^ Department of Neurology, Case Western Reserve University, Cleveland, Ohio, USA; ^4^ National Center for Regenerative Medicine, Case Western Reserve University, Cleveland, Ohio, USA; ^5^ The First Affiliated Hospital, Nanchang University, Nanchang, Jiangxi Province, The People's Republic of China; ^6^ Department of Pharmacology and EM Facility, Case Western Reserve University, Cleveland, Ohio, USA; ^7^ Department of Clinical and Experimental Medicine, Second University of Naples, Naples, Italy

**Keywords:** Alzheimer's disease, Aβ, prion protein, prion disease, brain, amyloid, electron microscopy

## Abstract

In transmission studies with Alzheimer's disease (AD) animal models, the formation of Aβ plaques is proposed to be initiated by seeding the inoculated amyloid β (Aβ) peptides in the brain. Like the misfolded scrapie prion protein (PrP^Sc^) in prion diseases, Aβ in AD shows a certain degree of resistance to protease digestion while the biochemical basis for protease resistance of Aβ remains poorly understood. Using *in vitro* assays, histoblotting, and electron microscopy, we characterize the biochemical and morphological features of synthetic Aβ peptides and Aβ isolated from AD brain tissues. Consistent with previous observations, monomeric and oligomeric Aβ species extracted from AD brains are insoluble in detergent buffers and resistant to digestions with proteinase K (PK). Histoblotting of AD brain tissue sections exhibits an increased Aβ immunoreactivity after digestion with PK. In contrast, synthetic Aβ40 and Aβ42 are soluble in detergent buffers and fully digested by PK. Electron microscopy of Aβ40 and Aβ42 synthetic peptides shows that both species of Aβ form mature fibrils. Those generated from Aβ40 are longer but less numerous than those made of Aβ42. When spiked into human brain homogenates, both Aβ40 and Aβ42 acquire insolubility in detergent and resistance to PK. Our study favors the hypothesis that the human brain may contain cofactor(s) that confers the synthetic Aβ peptides PrP^Sc^-like physicochemical properties.

## INTRODUCTION

Alzheimer's disease (AD) is the most common neurodegenerative disorder that affects 5% individuals over the age of 65 years and nearly half of people with age at 85 or older. It is the leading cause of dementia as it affects ~5.5 million of individuals in the US and ~24 million worldwide; moreover, the number of individual with AD has been predicted to duplicate in ~20 years [1]. Clinically, AD presents with progressive loss of memory, dementia, and cognitive impairment. Pathologically, it is characterized by the accumulation of extracellular amyloid-β (Aβ) deposits and intracellular hyperphosphorylated tau forming neurofibrillary tangles, dystrophic neurites, reactive microgliosis, oxidative damage and loss of neurons and synapses [2, 3]. According to the Amyloid cascade hypothesis, the formation of Aβ peptides following the cleavage of the Amyloid Precursor Protein (APP) is believed to be the first central event in the formation of larger aggregates [4, 5].

It has been recently shown that inoculation of transgenic mice expressing human APP with Aβ extracted from Alzheimer's patient brain causes onset and rapid progression of AD pathology [6, 7]. As a result, it has been proposed that the Aβ aggregates may act as a “seed” for the propagation and spread of the disease throughout the brain, in a way that resembles the propagation of the scrapie prion protein (PrP^Sc^) in subjects affected by prion diseases [8, 9]. However, the precise mechanism by which this takes place is not well understood. Similar to AD, the spread of aggregated PrP^Sc^ throughout the brain induces neurodegeneration and cell death. This observation has lead Aβ peptides to be described as either prions or prionoids [10, 11]. The discrepancy between these two descriptions lies in the dependence of Aβ peptides on cofactors for propagation and aggregate assembly. Recent findings suggest that Aβ peptides may in fact be bona-fide prions; however, co-factors present in human brain homogenate are likely to, at the very least, facilitate the formation of aggregates.

To further explore this phenomenon, we examined the biochemical “prion-like” properties of synthetic Aβ, Aβ extracted from AD brains, and synthetic Aβ spiked into brain homogenate. Indeed, certain biochemical hallmarks including detergent insolubility and protease resistance characteristics by prions are also germane to Aβ deposits found in brains of AD patients. Here we show that these biochemical characteristics are conferred upon synthetic Aβ when spiked into human brain homogenate.

## RESULTS

### Solubility of Aβ and PrP in AD

**Figure 1:**
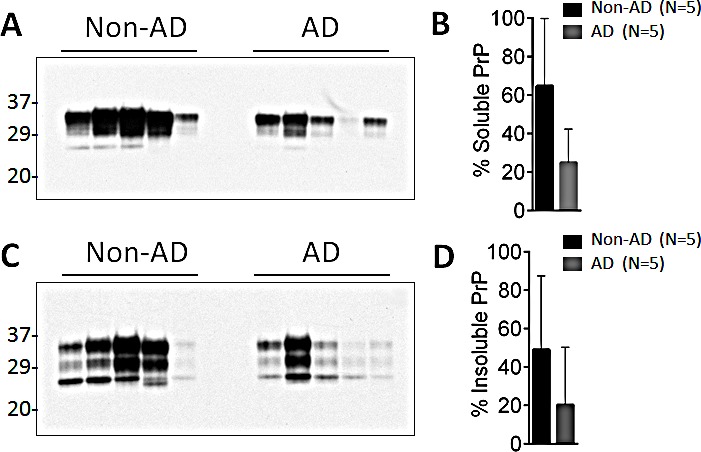
Levels of soluble and insoluble PrP from AD and non-AD patients 10% (w/v) brain homogenates (BH) from non-AD and AD affected patients were centrifuged at 100,000 x g for 1 h. After centrifugation, soluble PrP from the supernatant (A) and insoluble PrP from pellet (C) fractions were detected by western blotting with 3F4 in AD and non-AD brains. On average, the levels of soluble (B) and insoluble (D) PrP were higher in non-AD than in AD BH. Bar graphs are expressed as mean ± SEM of percent of PrP.

**Figure 2:**
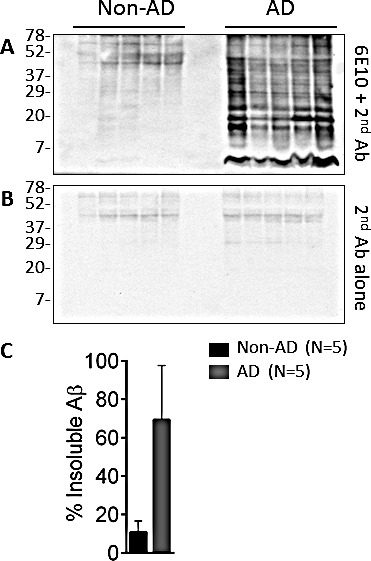
Levels of insoluble endogenous Aβ from AD and non-AD brains A: 10% (w/v) brain homogenates from non-AD and AD brains were centrifuged at 100,000 x g for 1 h. After centrifugation, insoluble Aβ from pellet fractions was detected in AD and non-AD brains with 6E10. B: The western blot showing non-specific background generated by the secondary antibody. C: The levels of insoluble Aβ from AD brains were ~6-fold higher than those of non-AD (P<0.002). Bar graphs are expressed as mean ± SEM of percent of insoluble Aβ.

The solubility of amyloid β (Aβ) and prion protein (PrP) in the brain of patients with Alzheimer's disease (AD) was analyzed by *in vitro* solubility assay. The levels of soluble and insoluble PrP in AD patients failed to show statistically significant differences with the ones observed in controls, despite the fact the levels of both soluble and insoluble PrP seemed more represented in the samples from the non-AD group (Fig. 1A through 1D). When the same cases were used to detect Aβ, insoluble Aβ was found to be significantly more abundant (~6-fold more) in AD patients than in controls (p = 0.009 <0.01) (Fig. 2). Soluble Aβ was virtually undetectable in both AD and non-AD by western blotting (data not shown), suggesting that most of Aβ is detergent-insoluble in the AD brain, consistent with our previous observation [12].

### PK-resistance of Aβ in the AD brain

**Figure 3:**
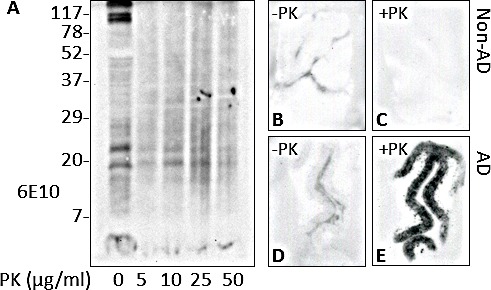
PK resistance of Aβ in AD brains A: Insoluble Aβ from the pellet fraction of AD brains was treated with increasing concentrations of proteinase K (PK). Endogenous brain Aβ exhibited PK resistance at least up to 50 μg/ml; antibody: 6E10. B through E: Histoblotting of Aβ from non-AD or AD brain samples. At variance with non-AD (B and C), the cryosection from AD brains (D and E) showed marked increased immunoreactivity after treatment with PK; antibody: 4G8.

To further investigate the physicochemical properties of the insoluble Aβ isolated from AD brain samples, the brain homogenates were then subjected to digestion with increasing levels of proteinase K (PK, ranged between 0 and 50 μg/ml) and immunoblotting with 6E10. The insoluble Aβ from AD brains was found to be resistant to PK digestion up to 50 μg/ml, as proved by the detection of monomeric and oligomeric Aβ species (Fig. 3A), thus sharing one of the main physicochemical properties of PrP^Sc^.

We next investigated the PK-resistance of Aβ in tissue sections of AD brains using histoblotting. Histoblot analysis with 4G8 antibody was performed to assess the presence and extent of Aβ accumulation in the brains of subjects with AD. The immunoreactivity observed in the cryosections was compared before and after treatment with PK. Interestingly, the detected signal in AD brains was very weak in PK-untreated sections (Fig. 3D) but appeared to increase dramatically after incubation with the enzyme (Fig. 3E). As expected, no immunoreactivity was observed in control sections from non-AD subjects, not even after PK-treatment (Fig. 3B and 3C).

### Solubility and PK-sensitivity of synthetic Aβ

**Figure 4:**
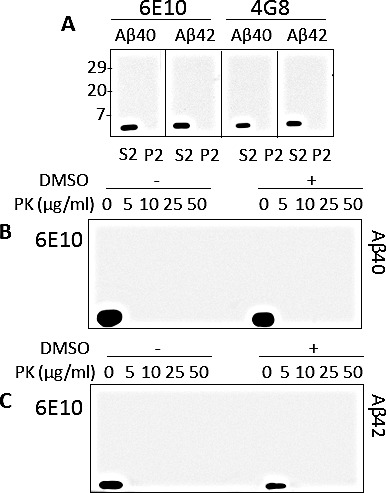
Detergent-solubility of synthetic Aβ A: Synthetic peptides Aβ40 and Aβ42 prepared in 1 x PBS were diluted in 2 x lysis buffer (pH 7.5) to a final concentration of 5 ng/ml and centrifuged at 100,000 x g for 1 h at 4°C. After centrifugation, aliquots from supernatant (S2) and pellet (P2) fractions were immunoblotted with 6E10 or 4G8 antibody. Both Aβ40 and Aβ42 were detected only in the S2 fraction. B and C: Aβ40 and Aβ42 prepared in 1X PBS were diluted in 1X lysis buffer with or without the solvent dimethylsulfoxide (DMSO), then digested with increasing concentrations of PK. Under these conditions the synthetic Aβ peptides were digested with the minimum amount of PK (5 μg/ml); antibody: 6E10.

To determine whether synthetic Aβ aggregates are similar to brain-derived ones in terms of physicochemical features, synthetic Aβ40 and Aβ42 peptides were solubilized in 1X PBS and further diluted in 2X lysis buffer at pH 7.5 to reach a final concentration of 5 ng/ml. After ultracentrifugation, Aβ peptides in supernatants (S2) and pellets (P2) were determined by western blotting. Aβ40 and Aβ42 were detectable by 6E10 and 4G8 only in the S2 but not in the P2 (Fig. 4A), demonstrating distinct behaviors from those displayed by Aβ from AD brains. Additionally, the two synthetic peptides were treated with PK (concentration ranging between 0 and 50 μg/ml) to investigate their sensitivity to the protease. No immunoreactivity with 6E10 was detected even in the Aβ40 or Aβ42 sample treated with the smallest amount of PK (5 μg/ml) (Fig. 4B and 4C). To evaluate if the sensitivity to PK digestion was partly determined by an inappropriate solubilization of Aβ40 and Aβ42, the synthetic peptides were dissolved in DMSO. The use of the solvent did not affect the results of the experiments, remarking the PK-sensitive nature of the two synthetic peptides (Fig. 4B and 4C).

### Electron microscopy of synthetic Aβ

**Figure 5:**
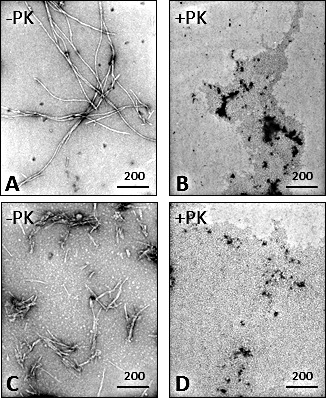
Electron microscopy of Aβ40 and Aβ42 peptides Aβ40 (A and B) and Aβ42 (C and D) fibrils were detectable only before PK digestion (−PK) (A and C). Aβ40 fibrils were longer but much less abundant than Aβ42 fibrils.

The detergent-soluble and PK-sensitive nature of the synthetic Aβ peptides examined above suggested that these peptides might not form aggregates. To exclude this possibility, the ultrastructure of the Aβ40 and Aβ42 peptides was determined by electron microscopy (EM). Surprisingly, both peptides were able to form mature fibrils but with heterogeneous features. Aβ42 generated numerous but short fibrils, whereas in Aβ40 preparation the fibrils were relatively scarce but much longer (Fig. 5A and 5C). It is possible that inability to detect Aβ40 and Aβ42 in PK-treated samples might be due to the limited amounts of the aggregated peptides, not sufficient for their detection by western blotting. To further rule out this possibility, PK-treated peptides were also examined by EM. While mature Aβ fibrils were readily observed, no fibrils or significant structures could be observed in the two peptide preparations after PK digestion, confirming that the synthetic Aβ40 and Aβ42 aggregates are PK-sensitive (Fig. 5B and 5D).

### Effect of brain homogenates on insolubility and PK-resistance of synthetic Aβ

**Figure 6:**
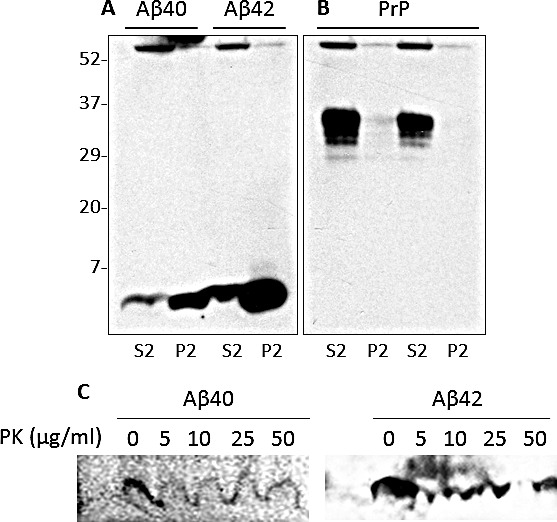
Effect of brain homogenate on detergent-insolubility and PK-resistance of synthetic Aβ A and B: After spiking Aβ40 and Aβ42 into a non-AD BH and ultra-centrifugation at 100,000 x g at 4°C for 1 h, aliquots from the supernatant (S2) and pellet (P2) fractions were examined for the presence of Aβ and PrP. The majority of synthetic Aβ40 or Aβ42 was detected in P2 fraction rather than in S2 by Western blotting with 6E10 (A). The same fractions probed with 3F4 showed that most of the PrP was soluble (B). C: Synthetic Aβ was spiked into a non-AD BH with no detectable endogenous Aβ, and then digested with increasing concentrations of PK. Under these conditions, synthetic Aβ40 and Aβ42 exhibited PK resistance up to 50 μg/ml. The blots were probed with the 6E10 antibody.

The inconsistence in physicochemical properties between synthetic and brain-derived Aβ40 and Aβ42 suggested that aggregation of the peptides themselves may not be sufficient to confer them detergent-insolubility and PK-resistance and that unknown brain factors could possibly participate in shaping the properties of the peptides. To determine the effect of brain homogenates on Aβ peptides, Aβ40 and Aβ42 were spiked into non-AD brain homogenates prepared in 1 x lysis buffer that did not display a significant level of endogenous Aβ. After ultracentrifugation, comparable volumes of S2 and P2 fractions were subjected to western blotting with 6E10 and 3F4. Aβ40 and Aβ42 were detected mostly in the P2, the fraction consisting of detergent-insoluble proteins (Fig. 6A). In contrast, PrP remained mostly soluble (Fig. 6B). To assess if this variation in the peptide solubility was associated with an increase in PK-resistance, the P2 fractions containing spiked peptides were digested with concentration of PK up to 50 μg/ml. Synthetic Aβ40 and Aβ42 spiked in brain homogenates all became PK-resistant, even in the samples treated with high enzyme concentrations (Fig. 6C).

**Figure 7:**
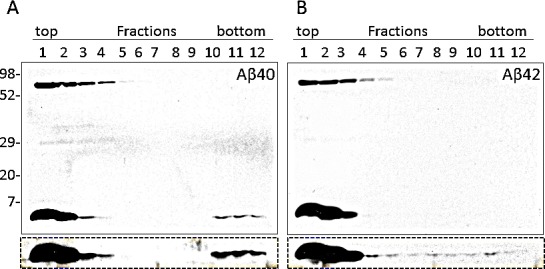
Velocity sedimentation in sucrose step gradients of synthetic Aβ Aβ40 and Aβ42 were spiked into the brain homogenate of transgenic mice expressing the human prion protein (HuPrP-Tg mice) and incubated at RT for 12 h. After sedimentation twelve fractions were collected from the top of the gradient. The levels of Aβ in each fraction were measured by western blotting with 6E10. Larger aggregates were observed in fractions 10-12 of the Aβ40 preparation (A), whereas they appeared to be minimal or absent in the Aβ42 preparation (B).

Furthermore, the two synthetic peptides were spiked into brain homogenates of transgenic mice expressing human PrP and incubated at room temperature for 12 hours. The samples were then loaded atop of discontinuous sucrose step gradients and centrifuged at 200,000 x g for 1h at 4°C. Twelve fractions were collected from the top of the gradients and immunoblotted with 6E10 (Fig. 7). The majority of Aβ40 and Aβ42 were found between fractions 1 and 3 at the top of the gradients. Notably, Aβ40 was also detected in significant amounts in fractions 10 to 12, indicating the presence of large Aβ40 aggregates (Fig. 7A). Compared to Aβ40 aggregates, smaller amounts of Aβ42 were detected in the bottom fractions (Fig. 7B).

## DISCUSSION

It is well-documented that detergent-insolubility and resistance to protease digestion are the two most dominant physicochemical characteristics of infectious PrP aggregates, and are widely used to differentiate between normal cellular PrP (PrP^C^) and the scrapie PrP (PrP^Sc^) associated with toxicity and infectivity [13]. Interestingly, recent studies demonstrate that amyloid β (Aβ) peptides, the neurotoxic aggregates accumulated in Alzheimer's brains, share these properties as well. Although these features are proposed to be closely associated with protein aggregation, the detailed molecular basis underlying the two physicochemical characteristics remains poorly understood. Efforts aimed to address this issue are especially important, given several lines of emerging evidence indicating that Aβ or α-synuclein aggregates, present in Alzheimer's or Parkinson's brains, could be infectious like prions [10].

Our current studies demonstrate that the physicochemical behaviors of the synthetic and brain-derived Aβ40/Aβ42 aggregates are different. The formers are detergent-soluble and PK-sensitive although they form aggregates, whereas the latters are detergent-insoluble and PK-resistant. The results agree with previously reported observations [14]. Moreover, we reveal in this study that the synthetic peptides acquire detergent-insolubility and protease-resistance after being spiked in the brain homogenates. Our study suggests that aggregation itself may not be sufficient to confer on proteins detergent-insolubility and PK-resistance, behaviors considered to be the key signatures for prion proteins to become infectious and/or neurotoxic. Our findings raise several issues as to the molecular basis underlying the pathogenic physicochemical properties of misfolding proteins, and implicate the role of other potential brain factors in shaping the physicochemical properties of the proteins involved in misfolding protein diseases including Alzheimer's and prion diseases.

PrP^C^ is the first cellular protein identified capable of forming the infectious isoform PrP^Sc^ [15]. The process of prion formation is believed to be associated with a conformational transition of the protein from α-helixes to β-sheets [13]. Along with this structural change, the pathologically-misfolded PrP^Sc^ acquires distinct physicochemical characteristics from its normal isoform PrP^C^, including detergent-insolubility, resistance to PK-digestion and infectivity. These structural, physicochemical and bioactive changes are proposed to be associated with aggregation of the proteins. Changes in conformation and oligomeric state of brain-derived PrP were mimicked with recombinant protein using different approaches [16, 17]. On the other hand, the physicochemical and bioactive changes have not been completely reproduced with recombinant PrP alone. For instance, the exact PK-resistant core fragment of typical PrP^Sc^ type 1 or type 2, found in the most common form of human prion diseases, has not been generated with recombinant human protein [18]. Interestingly, the PK-resistant PrP core fragment encompassing residues 97-231 similar to brain-derived PrP^Sc^ was generated by heating of the recombinant full-length hamster PrP in the presence of normal brain homogenate (a procedure termed annealing) [19, 20]. Importantly, recombinant hamster PrP annealed in the brain homogenates was reported to induce a new transmissible prion disease in wild-type mice [21]. Several studies claimed the generation of synthetic or recombinant prions in the absence of any mammalian cofactors [22-24]. However, it is worth noting that the infectivity of the recombinant PrP seeds has been always determined by inoculating the recombinant PrP aggregates into the brain. In this case, the possibility cannot be ruled out that the brain inoculated with the recombinant PrP aggregates may automatically provide necessary intrinsic cofactors for the recombinant PrP aggregates to form infectious PrP particles. This hypothesis seems to be favored by other studies in which other non-protein cofactors such as lipids and/or RNA were identified to be necessary for recombinant PrP to become infectious [25, 26].

The role of PK-resistance in PrP^Sc^-associated neurotoxicity remains controversial. In familial Gerstmann-Sträussler-Scheinker disease associated with P102L mutation, the presence of 8-kDa PK-resistant PrP peptide correlated with the presence of the amyloid plaques and/or of the PrP plaque-like deposits [27]. In contrast, significant amounts of the 21-kDa PK-resistant fragment were detected exclusively in brain regions showing spongiform degeneration. The same areas also displayed a punctate, ‘‘synaptic’’ type of immunoreactivity after immunostaining, similar to the most common subtype of sporadic Creutzfeldt-Jakob disease (sCJD) [27, 28]. Moreover, our recent study of familial human prion disease associated with 144-bp insertion mutations further confirmed the correlation between PK-resistant PrP^Sc^ and spongiform degeneration. However, it is important to point out that the same study also provided the convincing evidence that PK-sensitive, rather than the PK-resistant PrP^Sc^, may cause prion disease [29]. This is further supported by our identification of a novel human prion disease termed variably protease-sensitive prionopathy (VPSPr) whose hallmark is the presence of dominant PK-sensitive PrP^Sc^ [30-32].

Although the role of the PK-resistance of PrP^Sc^ in neurotoxicity is questionable, its role in the prion infectivity seems to be crucial. Again, transmission experiments performed with GSS cases associated with P102L mutation exhibited different outcomes associated with the 21-kDa or the 8-kDa PK-resistant fragment in the inocula. A successful transmission was achieved only when the 21-kDa PK-resistant PrP fragment was detected in the inoculum [33]. In contrast, the transmission failed when the GSS inoculum was characterized by the presence of 8-kDa PK-resistant PrP fragment alone, although striking PrP-amyloid deposition was observed in several mouse brains. Moreover, brains of these mice failed to transmit any neurological disease on passage, but PrP-amyloid deposition was again observed in the brains of the recipient mice [33]. Additionally, the low rate or even absence/failure of transmission of VPSPr observed by us [32] further confirmed that PK-resistance of PrP^Sc^ plays an important role in the transmissibility of misfolded protein.

In Alzheimer's disease, many lines of evidence have demonstrated that soluble Aβ oligomers rather than insoluble amyloid fibrils are neurotoxic [34]. The potential role of the PK-sensitive PrP^Sc^ in the pathogenesis of prion diseases revealed in recent studies [30-32] may be reminiscent to the soluble oligomeric Aβ in AD. Our present study indicates that similar to recombinant PrP, synthetic Aβ40 and Aβ42 peptides can acquire prion-like properties in the brain. Although the insoluble Aβ fibrils may not be neurotoxic, it is possible that they play an important role in spread of Aβ aggregates by prion-like self-propagation. The role of PK-resistant PrP^Sc^ in the pathogenesis of prion diseases, especially for the 8-kDa PK-resistant PrP fragment in GSS, may be similar to the insoluble Aβ fibrils in AD. For instance, the striking PrP-amyloid deposition observed in mouse brains inoculated with brain tissue from an 8-kDa PK-resistant fragment GSS case was associated with almost complete absence of prion disease transmission. Moreover, brains of these mice failed to transmit spongiform degeneration on to the next passage, whereas PrP-amyloid deposition was again observed in the brains of the recipient mice [33]. This unique type of condition is considered to be a proteinopathy instead of prion disease, caused by PrP amyloid that can seed amyloid accumulation in the brain. Recent exciting studies by Prusiner's group found that different conformations of synthetic or brain-derived Aβ aggregates are capable of defining different strains of the disease, which further draw parallels with prion diseases [35-37, 14].

## MATERIALS AND METHODS

### Reagents and antibodies

Phenylmethyl-sulfonyl fluoride (PMSF) was purchased from Sigma Chemical Co. (St. Louis, MO). The 3F4 antibody was used to probe PrP [38, 39]. The anti-Aβ monoclonal antibodies, including 6E10 (reactive to residues 1-16 of human Aβ) and 4G8 (reactive to residues 17-24 of human Aβ), were obtained from Signet (Signet Laboratories, Dedham, MA). The two synthetic Aβ40 and Aβ42 peptides were purchased from Sigma.

### Brain tissue

The protocol for the use of autopsy brain tissues was approved by the Institutional Review Board of Case Western Reserve University (Cleveland, OH). Frontal cortex tissues from cases of clinically and pathologically diagnosed AD (N = 5, ages 80 ± 11, range: 63-93 years) and normal controls (N = 5, ages 75 ± 8, range: 66-86 years) were used. They were obtained from the Case Brain Bank and the National Prion Disease Pathology Surveillance Center, respectively. The postmortem interval of these brain tissues was between 3 and 24 hours. Grey matter was dissected out and homogenized as described below.

### Histoblotting

Histoblot of AD (N = 3) and non-AD cases (N = 3) was prepared as previously described [40]. 10-12 μm cryosections were cut and transferred to nitrocellulose membranes that were previously dampened with lysis buffer (0.5% NP-40, 0.5% sodium deoxycholate, 100 mM NaCl, 10 mM EDTA, 100 mM Tris-HCl, pH 8.0). Membranes were thoroughly air dried, rehydrated for 30 min in Tris-buffer saline (TBS) containing 0.5% Tween 20 (TBS-T), blocked with 7% (w/v) non-fat dry milk in TBS-T for 25 minutes and washed in TBS-T. Proteins were either treated with 25 μg/ml PK in lysis buffer for 60 min at room temperature or left untreated. After 3 washes with TBS-T, the membranes were incubated for 25 minutes in 2M guanidine isothiocyanate. The membranes were then washed in TBS-T and blocked with 7% (w/v) non-fat dry milk/TBS-T for 25 min, before probing with 4G8 for 2 h at 37°C. After washing with TBS-T, the membranes were incubated with horseradish peroxidase (HRP) goat anti-mouse IgG secondary antibody for 1 hour at 37°C. The reaction was visualized with the chemiluminescence detection kit (ECL-Plus, GE-Amersham).

### Negative staining and electron microscopy

Aβ40 or Aβ42 in PBS was adsorbed onto carbon films supported on Formvar membrane coated nickel grids as described previously [12]. The excess buffered-protein solution was removed, and negatively stained with 2% uranylacetate. Grids were then washed by touching a buffer and the excess buffer blotted off immediately using a Whatman filter paper. Grids were then air-dried and kept at room temperature. Negatively stained specimens were observed by a JEOL 1200EX electron microscope (JEOL, Tokyo, Japan) with 80 kV of electron acceleration voltage.

### Sample preparation and *in vitro* solubility assay

The 10% (w/v) brain homogenates (BH) were prepared in 9 volumes of 1X lysis buffer containing detergents (10mM Tris, 150 mM NaCl, 0.5% Nonidet P-40, 0.5% deoxycholate, 5mM EDTA, pH 7.4) by homogenization of brain material with pestle on ice. Fresh BHs were centrifuged at 1,000 x g for 10 min at 4°C, the supernatant (S1) were collected while the pellets were discarded. The following steps were prepared as previously described [41]. The S1 were further centrifuged at 100,000 x g for 1 h at 4°C in order to generate the detergent -soluble (supernatant, S2) and -insoluble (pellet, P2) fractions. Pellets were re-suspended in lysis buffer.

### Velocity sedimentation in sucrose step gradients

Brain homogenates (20% w/v) were incubated with an equal volume of 2% Sarkosyl for 30 min on ice. Samples were loaded atop of a 10-60% sucrose gradient and centrifuged at 200,000 x g in the SW55 rotor for 1 h at 4°C as described with minor modification [42]. After centrifugation, the content of each tube was sequentially removed from the top to the bottom of the gradient and 12 fractions were collected.

### Proteinase K digestion and western blot analysis

Detergent insoluble pellet fractions from AD and normal brains were treated with 0, 5, 10, 25 and 50 μg/ml proteinase K (PK) for 1 hour at 37°C. Synthetic Aβ peptides were either prepared in lysis buffer or spiked into normal brain homogenates and incubated with PK 0 to 50 μg/ml as above. The enzymatic reaction was stopped by adding 2 mM of PMSF. Samples were mixed with an equal volume of 2X sample buffer (6% SDS, 5% β-mercaptoethanol, 20% glycerol, 4 mM EDTA, 125 mM Tris-HCl, pH 6.8), boiled for 10 min at 100 °C, loaded atop of a 15% Tris-glycine precast SDS-PAGE gels at 150 V for 80 min and transferred to PVDF membrane for 2 hours at 60 V. Membranes were blocked with 5% non-fat milk in TBS-T for 1 hour and incubated with the primary monoclonal antibodies anti-prion 3F4 (1:40,000), or anti-Aβ antibodies 6E10 (1:6,000) and 4G8 (1:6,000) for 2 hours at room temperature. After washing with TBS-T buffer, to remove the excess of antibody, membranes were incubated with a horseradish peroxidase-conjugated goat anti-mouse antibody (1:3,000) for 1 hour. Each membrane was developed by using a chemiluminescence substrate and visualized on Kodak Biomax MR and XAR films.

## References

[1] Mayeux R, Stern Y (2012). Epidemiology of Alzheimer disease. Cold Spring Harb Prespect Med.

[2] Holtzman DM, Morris JC, Goate AM (2011). Alzheimer's disease: the challenge of the second century. Sci Transl Med.

[3] Selkoe DJ (2001). Alzheimer's Disease: Genes, Proteins, and Therapy. Physiological Reviews.

[4] Hardy J, Selkoe DJ (2002). The amyloid hypothesis of Alzheimer's disease: progress and problems on the road to therapeutics. Science.

[5] Karran E, Mercken M, De Strooper B (2011). The amyloid cascade hypothesis for Alzheimer's disease: an appraisal for the development of therapeutics. Nat Rev Drug Discov.

[6] Kane MD, Lipinski WJ, Callahan MJ, Bian F, Durham RA, Schwarz RD, Roher AE, Walker LC (2000). Evidence for seeding of beta-amyloid by intracerebral infusion of Alzheimer brain extracts in beta-amyloid precursor protein-transgenic mice. J Neurosci.

[7] Watts JC, Giles K, Grillo SK, Lemus A, DeArmond SJ, Prusiner SB (2011). Bioluminescence imaging of Abeta deposition in bigenic mouse models of Alzheimer's disease. Proc Natl Acad Sci U S A.

[8] Langer F, Eisele YS, Fritschi SK, Staufenbiel M, Walker LC, Jucker M (2011). Soluble Aβ seeds are potent inducers of cerebral β-amyloid deposition. J Neurosci.

[9] Meyer-Luehmann M, Coomaraswamy J, Bolmont T, Kaeser S, Schaefer C, Kilger E, Neuenschwander A, Abramowski D, Frey P, Jaton AL, Vigouret JM, Paganetti P, Walsh DM, Mathews PM, Ghiso J, Staufenbiel M, Walker LC, Jucker M (2006). Exogenous induction of cerebral beta-amyloidogenesis is governed by agent and host. Science.

[10] Prusiner SB (2013). Biology and genetics of prions causing neurodegeneration. Annu Rev Genet.

[11] Aguzzi A (2009). Cell biology: Beyond the prion principle. Nature.

[12] Zou WQ, Xiao X, Yuan J, Puoti G, Fujioka H, Wang X, Richardson S, Zhou X, Zou R, Li S, Zhu X, McGeer PL, McGeehan J, Kneale G, Rincon-Limas DE, Fernandez-Funez P, Lee HG, Smith MA, Petersen RB, Guo JP (2011). Amyloid-beta42 interacts mainly with insoluble prion protein in the Alzheimer brain. J Biol Chem.

[13] Prusiner SB (1998). Prions. Proc Natl Acad Sci U S A.

[14] Stöhr J, Watts JC, Mensinger ZL, Oehler A, Grillo SK, DeArmond SJ, Prusiner SB, Giles K (2012). Purified and synthetic Alzheimer's amyloid beta (Aβ) prions. Proc Natl Acad Sci U S A.

[15] Prusiner SB (1982). Novel proteinaceous infectious particles cause scrapie. Science.

[16] Swietnicki W, Petersen R, Gambetti P, Surewicz WK (1997). pH-dependent stability and conformation of the recombinant human prion protein PrP(90-231). J Biol Chem.

[17] Cobb NJ, Apetri AC, Surewicz WK (2008). Prion protein amyloid formation under native-like conditions involves refolding of the C-terminal alpha-helical domain. J Biol Chem.

[18] Swietnicki W, Morillas M, Chen SG, Gambetti P, Surewicz WK (2000). Aggregation and fibrillization of the recombinant human prion protein huPrP90-231. Biochemistry.

[19] Bocharova OV, Breydo L, Salnikov VV, Gill AC, Baskakov IV (2005). Synthetic prions generated *in vitro* are similar to a newly identified subpopulation of PrPSc from sporadic Creutzfeldt-Jakob Disease. Protein Sci.

[20] Bocharova OV, Makarava N, Breydo L, Anderson M, Salnikov VV, Baskakov IV (2006). Annealing prion protein amyloid fibrils at high temperature results in extension of a proteinase K-resistant core. J Biol Chem.

[21] Makarava N, Kovacs GG, Bocharova O, Savtchenko R, Alexeeva I, Budka H, Rohwer RG, Baskakov IV (2010). Recombinant prion protein induces a new transmissible prion disease in wild-type animals. Acta Neuropathol.

[22] Legname G, Baskakov IV, Nguyen HO, Riesner D, Cohen FE, DeArmond SJ, Prusiner SB (2004). Synthetic mammalian prions. Science.

[23] Colby DW, Giles K, Legname G, Wille H, Baskakov IV, DeArmond SJ, Prusiner SB (2009). Design and construction of diverse mammalian prion strains. Proc Natl Acad Sci U S A.

[24] Kim JI, Cali I, Surewicz K, Kong Q, Raymond GJ, Atarashi R, Race B, Qing L, Gambetti P, Caughey B, Surewicz WK (2010). Mammalian prions generated from bacterially expressed prion protein in the absence of any mammalian cofactors. J Biol Chem.

[25] Wang F, Yang F, Hu Y, Wang X, Wang X, Jin C, Ma J (2007). Lipid interaction converts prion protein to a PrPSc-like proteinase K-resistant conformation under physiological conditions. Biochemistry.

[26] Deleault NR, Lucassen RW, Supattapone S (2003). RNA molecules stimulate prion protein conversion. Nature.

[27] Parchi P, Chen SG, Brown P, Zou W, Capellari S, Budka H, Hainfellner J, Reyes PF, Golden GT, Hauw JJ, Gajdusek DC, Gambetti P (1998). Different patterns of truncated prion protein fragments correlate with distinct phenotypes in P102L Gerstmann-Sträussler-Scheinker disease. Proc Natl Acad Sci U S A.

[28] Parchi P, Castellani R, Capellari S, Ghetti B, Young K, Chen SG, Farlow M, Dickson DW, Sima AA, Trojanowski JQ, Petersen RB, Gambetti P (1996). Molecular basis of phenotypic variability in sporadic Creutzfeldt-Jakob disease. Ann Neurol.

[29] Xiao X, Cali I, Dong Z, Puoti G, Yuan J, Qing L, Wang H, Kong Q, Gambetti P, Zou WQ (2013). Protease-sensitive prions with 144-bp insertion mutations. Aging (Albany NY).

[30] Gambetti P, Dong Z, Yuan J, Xiao X, Zheng M, Alshekhlee A, Castellani R, Cohen M, Barria MA, Gonzalez-Romero D, Belay ED, Schonberger LB, Marder K, Harris C, Burke JR, Montine T, Wisniewski T, Dickson DW, Soto C, Hulette CM, Mastrianni JA, Kong Q, Zou WQ (2008). A novel human disease with abnormal prion protein sensitive to protease. Ann Neurol.

[31] Zou WQ, Puoti G, Xiao X, Yuan J, Qing L, Cali I, Shimoji M, Langeveld JP, Castellani R, Notari S, Crain B, Schmidt RE, Geschwind M, Dearmond SJ, Cairns NJ, Dickson D, Honig L, Torres JM, Mastrianni J, Capellari S, Giaccone G, Belay ED, Schonberger LB, Cohen M, Perry G, Kong Q, Parchi P, Tagliavini F, Gambetti P (2010). Variably protease-sensitive prionopathy: a new sporadic disease of the prion protein. Ann Neurol.

[32] Zou WQ, Gambetti P, Xiao X, Yuan J, Langeveld J, Pirisinu L (2013). Prions in Variably Protease-Sensitive Prionopathy: An Update. Pathogens.

[33] Piccardo P, Manson JC, King D, Ghetti B, Barron RM (2007). Accumulation of prion protein in the brain that is not associated with transmissible disease. Proc Natl Acad Sci U S A.

[34] Walsh DM, Selkoe DJ (2007). A beta oligomers - a decade of discovery. J Neurochem.

[35] Aguzzi A (2014). Neurodegeneration: Alzheimer's disease under strain. Nature.

[36] Watts JC, Condello C, Stöhr J, Oehler A, Lee J, DeArmond SJ, Lannfelt L, Ingelsson M, Giles K, Prusiner SB (2014). Serial propagation of distinct strains of Aβ prions from Alzheimer's disease patients. Proc Natl Acad Sci U S A.

[37] Stöhr J, Condello C, Watts JC, Bloch L, Oehler A, Nick M, DeArmond SJ, Giles K, DeGrado WF, Prusiner SB (2014). Distinct synthetic Aβ prion strains producing different amyloid deposits in bigenic mice. Proc Natl Acad Sci U S A.

[38] Kascsak RJ, Rubenstein R, Merz PA, Tonna-DeMasi M, Fersko R, Carp RI, Wisniewski HM, Diringer H (1987). Mouse polyclonal and monoclonal antibody to scrapie-associated fibril proteins. J Virol.

[39] Zou WQ, Langeveld J, Xiao X, Chen S, McGeer PL, Yuan J, Payne MC, Kang HE, McGeehan J, Sy MS, Greenspan NS, Kaplan D, Wang GX, Parchi P, Hoover E, Kneale G, Telling G, Surewicz WK, Kong Q, Guo JP (2010). PrP conformational transitions alter species preference of a PrP-specific antibody. J Biol Chem.

[40] Taraboulos A, Jendroska K, Serban D, Yang SL, DeArmond SJ, Prusiner SB (1992). Regional mapping of prion proteins in brain. Proc Natl Acad Sci U S A.

[41] Cali I, Castellani R, Yuan J, Al-Shekhlee A, Cohen ML, Xiao X, Moleres FJ, Parchi P, Zou WQ, Gambetti P (2006). Classification of sporadic Creutzfeldt-Jakob disease revisited. Brain.

[42] Yuan J, Xiao X, McGeehan J, Dong Z, Cali I, Fujioka H, Kong Q, Kneale G, Gambetti P, Zou WQ (2006). Insoluble aggregates and protease-resistant conformers of prion protein in uninfected human brains. J Biol Chem.

